# Differential regulation of proliferation and neuronal differentiation in adult rat spinal cord neural stem/progenitors by ERK1/2, Akt, and PLCγ

**DOI:** 10.3389/fnmol.2013.00023

**Published:** 2013-08-27

**Authors:** Wai Si Chan, Alexandra Sideris, Jhon J. Sutachan, Jose V. Montoya G, Thomas J. J. Blanck, Esperanza Recio-Pinto

**Affiliations:** ^1^Department of Anesthesiology, New York University Langone Medical CenterNew York, NY, USA; ^2^Departamento de Nutrición y Bioquímica, Pontificia Universidad JaverianaBogotá, Colombia

**Keywords:** Neuronal differentiation, ERK1/2, Akt, PLCγ, progenitors, spinal cord

## Abstract

Proliferation of endogenous neural stem/progenitor cells (NSPCs) has been identified in both normal and injured adult mammalian spinal cord. Yet the signaling mechanisms underlying the regulation of adult spinal cord NSPCs proliferation and commitment toward a neuronal lineage remain undefined. In this study, the role of three growth factor-mediated signaling pathways in proliferation and neuronal differentiation was examined. Adult spinal cord NSPCs were enriched in the presence of fibroblast growth factor 2 (FGF2). We observed an increase in the number of cells expressing the microtubule-associated protein 2 (MAP2) over time, indicating neuronal differentiation in the culture. Inhibition of the *m*itogen-activated protein kinase or extracellular signal-regulated kinase (ERK) *k*inase 1 and 2/ERK 1 and 2 (MEK/ERK1/2) or the phosphoinositide 3-kinase (PI3K)/Akt pathways suppressed active proliferation in adult spinal cord NSPC cultures; whereas neuronal differentiation was negatively affected only when the ERK1/2 pathway was inhibited. Inhibition of the phospholipase Cγ (PLCγ) pathway did not affect proliferation or neuronal differentiation. Finally, we demonstrated that the blockade of either the ERK1/2 or PLCγ signaling pathways reduced neurite branching of MAP2+ cells derived from the NSPC cultures. Many of the MAP2+ cells expressed synaptophysin and had a glutamatergic phenotype, indicating that over time adult spinal cord NSPCs had differentiated into mostly glutamatergic neurons. Our work provides new information regarding the contribution of these pathways to the proliferation and neuronal differentiation of NSPCs derived from adult spinal cord cultures, and emphasizes that the contribution of these pathways is dependent on the origin of the NSPCs.

## Introduction

Early studies of the adult mammalian central nervous system identified the presence of neural stem/progenitor cells (NSPCs) in the adult spinal cord (Weiss et al., 1996; Shihabuddin et al., 1997). Subsequent research showed that proliferation of the adult NSPCs are stimulated in animal models of spinal cord injury (Johansson et al., 1999; Horner et al., 2000; Yamamoto et al., 2001; Danilov et al., 2006). Some studies reported that the fate of the proliferating NSPCs is restricted to glial cell types *in vivo* due to the inhibitory microenvironment of the adult spinal cord (Johansson et al., 1999; Shihabuddin et al., 2000; Horky et al., 2006; Yang et al., 2006; Barnabe-Heider et al., 2010). Nonetheless, the consensus is that adult spinal cord NSPCs are intrinsically multipotent (i.e., they can also generate neurons), as demonstrated by transplantation studies and neurosphere assays (Shihabuddin et al., 2000; Yamamoto et al., 2001). In fact, neurogenesis in the adult spinal cord has been detected *in vivo* in both pathological (Danilov et al., 2006; Vessal et al., 2007) and normal conditions (Shechter et al., 2007, 2010). However, as we gain more insight into the existence and implications of neurogenesis in the adult spinal cord, the question remains as to how these adult spinal cord NSPCs are regulated. A better understanding of the basic biology of these NSPCs will facilitate future attempts to manipulate these cells under pathological conditions.

Unlike in the adult spinal cord, the occurrence of neurogenesis in the adult hippocampus has been firmly established (Alvarez-Buylla and Garcia-Verdugo, 2002; Ming and Song, 2005, 2011). Astrocytes from this brain region have been shown to induce neurogenesis of adult hippocampal NSPCs via the Wnt signaling pathway (Song et al., 2002; Lie et al., 2005). Furthermore, diffusible factors from the neurovascular niche are reported to stimulate neurogenesis in the adult subventricular zone (Palmer et al., 2000; Shen et al., 2004, 2008). On the other hand, astrocytes from the adult spinal cord do not promote neurogenesis in culture (Song et al., 2002).

Growth factors, such as fibroblast growth factor 2 (FGF2), epidermal growth factor, nerve growth factor, and vascular endothelial growth factor, can elicit a range of cellular responses including cell proliferation, migration, differentiation, and cell death through various classes of receptor tyrosine kinases (RTKs) (Hubbard and Till, 2000). The activation of these different RTKs in turn induces the activation of several signal transduction pathways including the *m*itogen-activated protein kinase or extracellular signal-regulated kinase (ERK) *k*inase 1 and 2/ERK 1 and 2 (MEK/ERK1/2), phosphoinositide 3-kinase (PI3K)/Akt, and phospholipase Cγ (PLCγ) pathways (Huang et al., 2001; Mason, 2007). Much effort has gone into elucidating the signaling cascades involved in the neurogenesis of adult hippocampal NSPCs. For instance, both the MEK/ERK1/2 and PI3K/Akt pathways have been shown to be involved in regulating proliferation and self-renewal (Peltier et al., 2007; Ma et al., 2009), while the PLCγ pathway is reported to be essential for neuronal differentiation (Ma et al., 2009) of adult hippocampal NSPCs. Yet the role of these signaling pathways in adult spinal cord NSPCs has not been established. In this study, our aim was to assess the potential contribution of the MEK/ERK1/2, PI3K/Akt, and PLCγ pathways in proliferation and neuronal differentiation of NSPCs derived from the adult rat spinal cord culture. Our findings reinforce the idea that the contribution of signaling pathway activation in proliferation and neuronal differentiation is unique in different cell types and is dependent on the source of NSPCs.

## Results

### Enrichment of adult spinal cord-derived NSPCs

**Figure 1:**
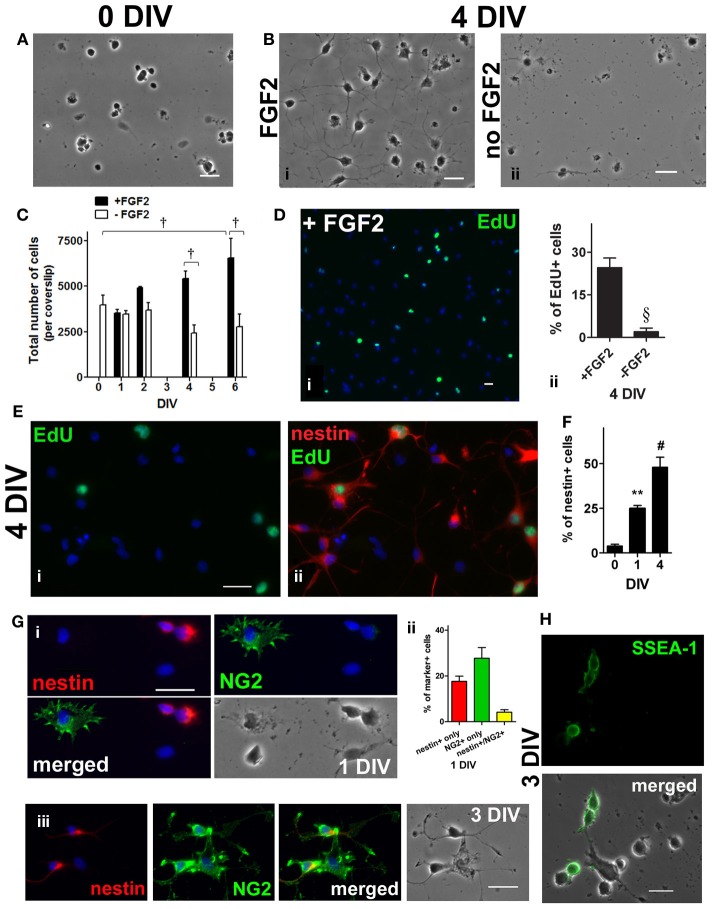
**Enrichment of adult rat spinal cord neural stem/progenitor cells (NSPCs) in culture. (A)** Dissociated adult spinal cord cells, 1 h after isolation. **(B)** Spinal cord cells after 4 days in FGF2 (i) or in basal medium (ii). **(C)** Adult spinal cord cells proliferated as the total cell number per coverslip increased over time in culture medium containing FGF2 [all the cells in each coverslip were counted, *n* = 3 coverslips; ^†^*P* < 0.001 compared with 0 days *in vitro* (DIV), and between 4 DIV and 6 DIV ± FGF2, One-Way ANOVA Dunnett's multiple comparison post-test]. **(D)** A thymidine analogue, EdU, was added to culture medium at 4 DIV, 6 h before fixation. Proliferating (actively dividing) cells were EdU-labeled (green) during the 6 h pulse (i). Blue indicates Hoechst-stained nuclei. (ii) Quantification of the percentage of EdU+ cells (10 random fields with a 40× objective per coverslip were analyzed, *n* = 3 coverslips; ^§^*P* < 0.005, unpaired *t*-test). **(E)** Co-labeling of EdU and nestin showing that some of the NSPCs (4 DIV) were actively dividing. EdU was added to culture medium for only 6 h before fixation at 4 DIV. (i) Proliferating (EdU+) cells (green) during the 6 h pulse. (ii) Merged picture of the green and red channels showing EdU (green) and nestin (red) staining. Blue indicates Hoechst-stained nuclei. **(F)** Quantification of the percentage of nestin+ cells showing that the population of NSPCs was enriched in culture medium containing FGF2 over time (20 random fields with a 40× objective per coverslip were analyzed, *n* = 4 coverslips; ^**^P < 0.01, ^#^*P* ≤ 0.0001, One-Way ANOVA Dunnett's multiple comparison post-test). **(G)** Co-labeling of NG2 chondronitin sulfate proteoglycan (green) and nestin (red) at 1 DIV (i) and 3 DIV (iii). At 1 DIV, most of the NG2+ cells had distinct morphology from nestin+ cells; while cells coexpressing both markers were observed at 1 and 3 DIV. Blue indicates Hoechst-stained nuclei. (ii) Quantification of the percentage of nestin+, NG2+, and nestin+/NG2+ cells at 1 DIV (20 random fields with a 40× objective per coverslip were analyzed, *n* = 4 coverslips). **(H)** At 3 DIV, stage-specific embryonic antigen 1 (SSEA-1) (green), a cell surface carbohydrate epitope found on uncommitted NSPCs, appeared to localize to the plasma membrane of cells with undifferentiated morphology. All scale bars: 20 μm.

The enriched adult spinal cord NSPCs were obtained by using a cell isolation protocol that yielded about 1% of the total number of cells in the spinal cord (see Materials and Methods) (Figure 1A); these cells could proliferate as neurospheres (not shown) or in adherent cultures in medium containing FGF2 (Figure 1B). Adherent cultures were used because we could assess proliferation in a shorter time frame thus avoiding possible phenotypic changes and/or selection of a subpopulation of NSPCs as a result of long term cultivation and passaging. In addition, adherent cultures in a single layer also facilitated identification and quantification of immunolabeled cells with various markers. Under such condition, the total cell number increased by 1.7-fold by 6 days *in vitro* (DIV) when compared to 0 DIV (Figure 1C). A thymidine analogue, EdU, was added to the culture to pulse-label those cells undergoing cell division at 4 DIV, 6 h prior to fixation (Figure 1D,i). During the 6-h pulse of EdU-labeling, 25% of the cells were EdU+ (Figure 1D,ii). In addition, co-labeling of EdU and nestin, used here as an adult NSPC marker (Lendahl et al., 1990; Johansson et al., 1999; Namiki and Tator, 1999; Fu et al., 2005), revealed that 32 ± 5% (*n* = 4) of nestin+ cells were EdU+ and that 46 ± 12% (*n* = 4) of EdU+ cells were nestin+ (Figure 1E). Immunostaining with nestin at 0, 1, and 4 DIV showed an increase of nestin+ cells over time; with a 12-fold increase in percentage by 4 DIV (Figure 1F). Cells were also labeled with another progenitor marker, neural-glial antigen 2 (NG2), as studies have shown that NG2+ cells are proliferative and can give rise to neurons (Belachew et al., 2003; Tamura et al., 2007; Guo et al., 2010). At 1 DIV 28% of the cells were immunopositive for NG2 (Figure 1G,i,ii) and 19% of the nestin+ cells were also NG2+ at 1 DIV (Figure 1G,ii). NG2 and nestin coexpressing cells were also detected at 3 DIV (Figure 1G,iii). The cells that were NG2+ were stellate-like, with short processes extending from all directions; and the cells that were only nestin+ were mostly rounded/spindle-shaped. The morphology of the cells co-labeled with nestin and NG2 was similar to that of the nestin+ cells (Figure 1G,iii). Based on the cell morphology, some of the EdU+ cells that were not immunopositive for nestin were likely NG2+ (not shown). Stage-specific embryonic antigen 1 (SSEA-1), a marker for undifferentiated NSPCs (Capela and Temple, 2002; Sabourin et al., 2009), was observed in about one-tenth of the cells in culture at 3 DIV (Figure 1H). Taken together, the expression of these markers demonstrates the presence of uncommitted NSPCs at 1–4 DIV.

### Neuronal differentiation in adult spinal cord NSPC cultures

**Figure 2:**
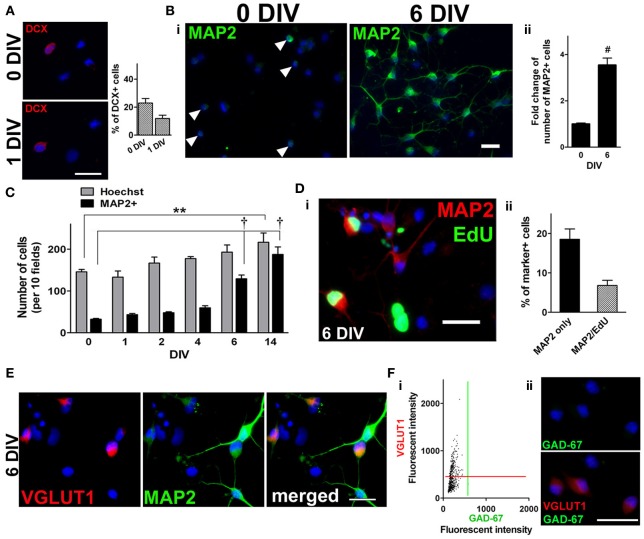
**Neuronal differentiation in adult spinal cord NSPC cultures. (A)** Immunostaining for doublecortin (DCX) at 0 DIV (top left panel) and 1 DIV (bottom left panel). Blue indicates Hoechst-stained nuclei. (Right panel) Quantification of the percentage of DCX+ cells at both time points (20 random fields with a 40× objective per coverslip were analyzed, *n* = 4 coverslips). **(B)** i, Microtubule-associated protein 2 (MAP2) staining (green) of adult rat spinal cord cultures at 0 DIV, arrowheads denote MAP2+ cells (left), and at 6 DIV in culture medium containing FGF2 (right). Blue indicates Hoechst-stained nuclei. (ii) Fold change of the number of MAP2+ cells at 6 DIV (*n* = 13 coverslips from five experiments; ^#^*P* < 0.0001, unpaired *t*-test). The data was normalized to the mean total number of cells at 0 DIV. **(C)** As the population of NSPCs was enriched, many of the cells differentiated toward a neuronal lineage as the number of MAP2+ cells increased over time (10 random fields with a 40× objective per coverslip were analyzed, *n* = 3 coverslips; ^**^P < 0.01, ^†^*P* < 0.001, One-Way ANOVA Dunnett's multiple comparison post-test). **(D)** i, Examples of newly generated MAP2+ cells (*green* EdU+ nuclei and *red* MAP2+ somata) by 6 DIV. As EdU was added to the culture medium for a 24-h period at 5 DIV, only those cells that were dividing then were labeled green. Blue indicates Hoechst-stained nuclei. (ii) Quantification of the percentage of EdU+/MAP2+ cells at 6 DIV (10 random fields per coverslips were analyzed, *n* = 8 coverslips from three experiments). **(E)** Immunostaining for vesicular glutamate transporter 1 (VGLUT1) and MAP2 revealed that 42% of the MAP2+ neurons had a glutamatergic phenotype (*n* = 539 cells). Cultures were counterstained with Hoechst (blue). **(F)** The majority (96%) of VGLUT1+ cells were negative for glutamic acid decarboxylase 67 (GAD-67) at 6 DIV. (i) A scatter plot of the fluorescent intensity of each individual cell (*n* = 554 cells). Red and green lines denote “cut-offs” for VGLUT1 and GAD-67 immunoreactivity, respectively. Cells with intensity values above each line were considered positively stained. (ii) Example of VGLUT1+/GAD-67- cells at 6 DIV. Cells were counterstained with Hoechst (blue). Scale bars: 20 μm.

Maintaining the expanded population of adult spinal cord NSPCs in medium containing FGF2 eventually resulted in neuronal differentiation. At 0 DIV and 1 DIV, about 23 and 12% of the cells were doublecortin-positive (DCX+) immature neurons (Figure 2A) (the remainder of the cells are most likely nestin+ and/or NG2+ cells, see Figure 1G,ii). Over time, there was an increase in the number of cells expressing the neuronal marker, microtubule-associated protein 2 (MAP2) (Figure 2B i). The number of MAP2+ cells increased by nearly 4-fold by 6 DIV (Figure 2B,ii) and about 6-fold by 14 DIV (Figure 2C). To demonstrate that some of these MAP2+ cells were newly generated, at 5 DIV proliferating cells were labeled with a 24 h EdU pulse, and the culture was immunolabeled for MAP2 at 6 DIV. After the pulse of EdU, about a quarter of all MAP2+ cells were found to be EdU+ (Figure 2D,i,ii). When FGF2 was not added to the culture medium there was no increase in the number of MAP2+ cells nor an increase in total cell number (not shown). Antibodies against vesicular glutamate transporter 1 (VGLUT1) and glutamic acid decarboxylase 67 (GAD-67) were used to further characterize the neuronal phenotype of these newly generated MAP2+ cells (i.e., whether they were excitatory or inhibitory neurons). We found that 42% of all the MAP2+ cells were VGLUT1+ (Figure 2E). Moreover, 96% of all the VGLUT1+ cells were negative for GAD-67 (Figure 2F), suggesting that adult spinal cord-derived NSPCs differentiate toward neurons with an excitatory phenotype.

### Proliferation of adult spinal cord NSPC cultures requires activation of ERK1/2 and Akt

**Figure 3:**
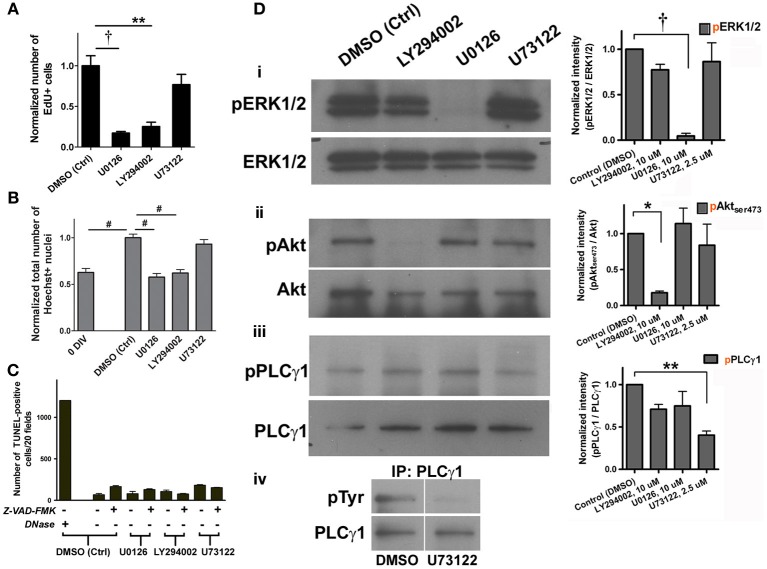
**Cell proliferation in adult spinal cord NSPC cultures requires activation of ERK1/2 and Akt. (A)** Quantification of the normalized number of EdU+ cells showed that inhibition of ERK1/2 (with MEK inhibitor, U0126, 10 μ M) and Akt (with PI3K inhibitor, LY294002, 10 μ M) activation suppressed proliferation of adult spinal cord cells at 4 DIV; inhibition of PLCγ activation (with U73122, 2.5 μ M) had no effect (10 random fields with a 40× objective per coverslip were analyzed, *n* = 3 coverslips; ^**^P < 0.01, ^†^*P* < 0.001, One-Way ANOVA Bonferroni's multiple comparison post-test). EdU was added to culture medium 6 h before fixation. **(B)** Normalized total number of Hoechst-stained nuclei to the DMSO control grouped showed that inhibition of both ERK1/2 and Akt activation decreased total number of cells at 4–6 DIV (*n* = 9 coverslips, except LY294002: *n* = 8 coverslips, and U73122: *n* = 6 coverslips, from three experiments; ^#^*P* ≤ 0.0001, One-Way ANOVA Bonferroni's multiple comparison post-test). All values were normalized to the DMSO control group in each experiment. **(C)** Quantification of the number of TUNEL-positive cells at 4 DIV to determine cell survival after treatment with each of the inhibitors. None of the treatment significantly changed cell survival as compared to DMSO control, even with the addition of Z-VAD-FMK (20 μ M), a general caspase inhibitor (20 random fields with a 20× objective per coverslip were analyzed, *n* = 3 coverslips for all except for the first bar (DNase as a positive control for the TUNEL assay): *n* = 2 coverslips). **(D)** i–iii, Western blot analysis showed that the MEK/ERK1/2, PI3K/Akt, and PLCγ pathways were activated in adult spinal cord cultures (6 DIV). The right panel shows the ratios of phosphorylated protein to total protein (pERK1/2 to ERK1/2, pAkt to Akt, and pPLCγ 1 to PLCγ 1) in the presence of each of the inhibitors. Each of the pharmacological inhibitors specifically blocked phosphorylation of its respective target protein when exposed to FGF2. Quantified results of the blots generated using the ImageJ software (*n* = 3 experiments; ^*^P < 0.05, ^**^P < 0.01, ^†^*P* < 0.001, One-Way ANOVA Bonferroni's multiple comparison post-test). (iv) Total PLCγ protein was pulled down and then the membrane was blotted for tyrosine residues. Upper right corner shows that PLCγ phosphorylation was blocked by 2.5 μ M of U73122.

To examine which of the signaling pathways might be important for proliferation, specific pharmacological inhibitors were each applied to the culture for 4 days, to which EdU was also added for 6 h on day 4. We found that treatment with 10 μ M U0126 (MEK1/2 inhibitor) or 10 μ M LY294002 (PI3K inhibitor) significantly reduced the number of EdU+ cells compared to the vehicle control (Figure 3A). This indicates that the activation of both MEK/ERK and PI3K/Akt signaling pathways contributed to the proliferation of adult spinal cord-derived NSPCs. Consistent with this interpretation, we found that both treatments with U0126 and LY294002 for 4–6 days resulted in the suppression of an increase in the total cell number (total Hoechst-stained nuclei) (Figure 3B). In contrast, there was no indication that the PLCγ pathway was involved in NSPC proliferation, as 2.5 μ M U73122 (PLCγ inhibitor) did not significantly affect the number of EdU+ cells or the total cell number when compared to the vehicle control (Figures 3A,B). TUNEL analysis was performed to address whether the inhibitors affected cell survival. None of the inhibitors significantly increased the number of TUNEL-positive cells compared to the vehicle control. Z-VAD-FMK, a general caspase inhibitor, did not decrease the number of TUNEL-positive cells in the presence of any of the inhibitors (Figure 3C), indicating that the decrease in EdU+ cell and total cell number as a result of the treatment with U0126 or LY294002 was not due to apoptosis. The specificity of each of the pharmacological inhibitors used was verified in the adult spinal cord culture enriched in NSPCs by Western blot analysis. By using antibodies specific for ERK1/2, Akt, and PLCγ 1 and their phosphorylated isoforms, the ratio of phosphorylated protein to total protein (pERK1/2 to ERK1/2, pAkt to Akt, and pPLCγ 1 to PLCγ 1) in the presence of each of the inhibitor was measured. We confirmed that, at the concentrations used in the cultures, each of the three inhibitors specifically and significantly blocked the phosphorylation of ERK1/2, Akt, and PLCγ 1 (Figure 3D).

### Neuronal differentiation in adult spinal cord NSPC cultures is ERK1/2-dependent

**Figure 4:**
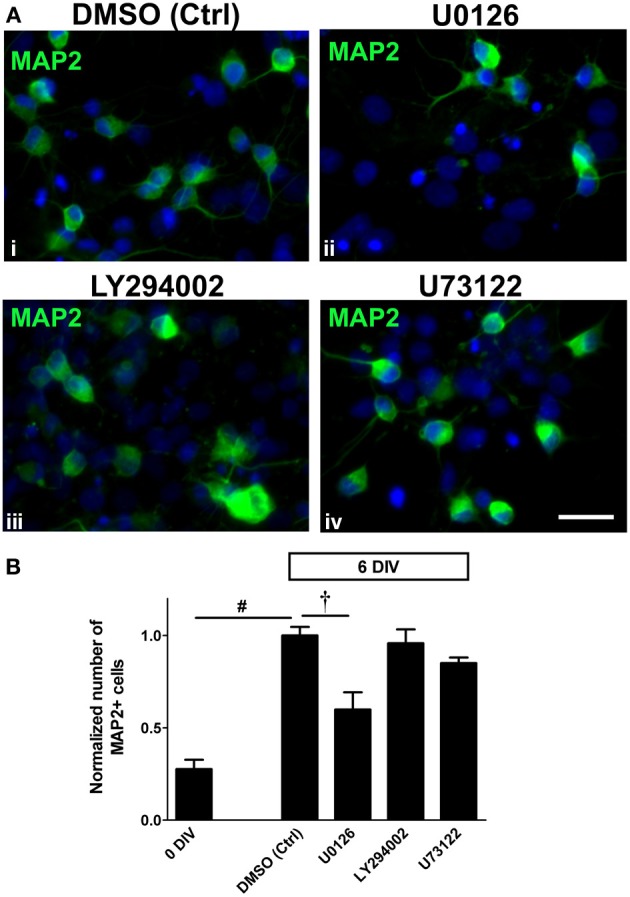
**Neuronal differentiation in adult spinal cord NSPC cultures is ERK1/2-dependent. (A)** Immunostaining for MAP2 in adult spinal cord NSPC cultures after 6 DIV in vehicle control (DMSO) (i), U0126 (10 μ M) (ii), LY294002 (10 μ M) (iii), and U73122 (2.5 μ M) (iv). Compared to the vehicle control (i), the number of MAP2+ cells was noticeably less when the cells were treated with the MEK inhibitor, U0126 (ii). Blue represents Hoechst-stained nuclei. Scale bar: 20 μm. **(B)** Quantification of the normalized number of MAP2+ cells in adult spinal cord NSPC cultures (6 DIV). By 6 days, there was a similar increase in MAP2+ cell number as shown in Figure 2. Only U0126 (which inhibited ERK1/2 activation) significantly inhibited the increase of the number of MAP2+ cells after 6 days. LY294002 (10 μ M) and U73122 (2.5 μ M) had no significant effect on neuronal differentiation (10 random fields with a 40× objective per coverslip were analyzed, *n* = 6 coverslips, except LY294002: *n* = 5 coverslips and U73122: *n* = 3 coverslips; ^†^*P* < 0.001, ^#^*P* ≤ 0.0001, One-Way ANOVA Bonferroni's multiple comparison post-test). All values are normalized to the DMSO control in each experiment.

To evaluate whether each of the inhibitors might influence the neuronal differentiation potential of NSPCs, we examined the number of MAP2+ neurons in each treatment. We found that neuronal differentiation of the adult spinal cord-derived NSPCs was only suppressed by treatment with 10 μ M U0126, inhibitor of MEK and therefore ERK1/2 activation, such that there was 40% less MAP2+ neurons as compared to the DMSO control by 6 DIV (Figures 4A i,ii, B). Inhibition of the PI3K/Akt and PLCγ pathways had no significant effect on the number of cells expressing MAP2 (Figures 4A i,iii,iv, B). Taken together, this indicates that the MEK/ERK1/2 pathway had a crucial role in neuronal differentiation.

### Neurite branching of MAP2+ neurons derived from adult spinal cord NSPC cultures requires activation of ERK1/2 and PLCγ

**Figure 5:**
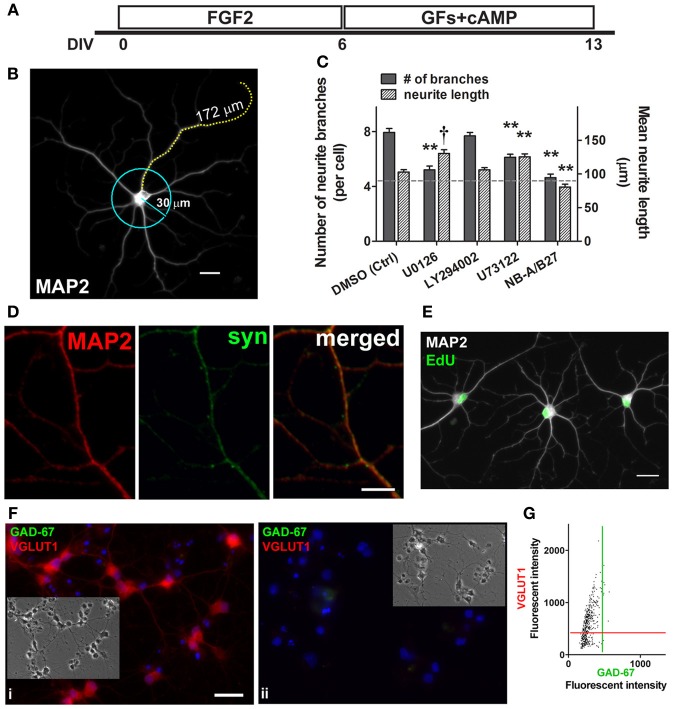
**Neurites branching requires ERK1/2 and PLCγ activation; some neurons form synapses and are glutamatergic. (A)** Schematic of experimental protocol used in the neurite studies. FGF2 was present in culture to expand nestin+ NSPCs (0–6 DIV). Culture medium containing FGF2 was then replaced with BDNF, GDNF (GFs), and cAMP to promote further differentiation of MAP2+ neurons (6–13 DIV). **(B)** A typical MAP2+ neuron derived from an adult spinal cord NSPC culture (13 DIV). The number of neurite branches of each MAP2+ neuron in each treatment was analyzed by scoring those branches that intersected the perimeter of a 30 μm-circle. The mean neurite length was quantified by measuring the length of the longest neurite of each MAP2+ neuron in each treatment. Scale bar: 20 μm. **(C)** Quantification of neurite branching (solid bars) and neurite length (patterned bars) of MAP2+ cells (13 DIV). Dotted gray line represents average measurements (left Y-axis: number of neurite branches; right Y-axis: mean neurite length) at 6 DIV before growth factor treatment. By 13 DIV, added growth factors increased neurite branches in DMSO control from 4.0 ± 0.4 (at 6 DIV) to 7.9 ± 0.3 (at 13 DIV) (*n* = 25 and 87 cells, respectively). The number of branches in NB-A/B27 control with no added growth factor (4.6 ± 0.3 at 13 DIV; *n* = 94 cells) remained similar to that at 6 DIV (4.0 ± 0.4; *n* = 25 cells). Neurite branching of MAP2+ neurons was significantly suppressed when the activation of ERK1/2 and PLCγ was respectively blocked by 10 μ M U0126 (5.2 ± 0.3 at 13 DIV; *n* = 83 cells) and 2.5 μ M U73122 (6.1 ± 0.2 at 13 DIV; *n* = 96 cells). The blockage of the activation of ERK1/2 and PLCγ resulted in an increase in the neurite length of the MAP2+ neurons (U0126: 130.4 ± 5.6 μm, *n* = 83 cells; U73122: 125.4 ± 4.4 μm, *n* = 96 cells). PI3K inhibitor, LY294002 (10 μ M), had no effect on neurite branching or elongation (*n* = 98 cells). Inhibitor(s) or vehicle control (DMSO) was added to culture from 6 DIV, except in the last condition (NB-A/B27) in which the culture was maintained in basal medium only with no growth factor treatment (For each condition, three coverslips were analyzed; ^**^P < 0.01, ^†^*P* < 0.001, One-Way ANOVA Bonferroni's multiple comparison post-test). **(D)** MAP2+ (red) neurons displayed synapse formation with one another, as evidenced by co-labeling with anti-synaptophysin (syn, green) (13 DIV). Scale bar: 5 μm. **(E)** Evidence of newly generated neurons (cells with *green* EdU+ nuclei and *white* MAP2+ labeling) at 13 DIV with processes that came in contact with neighboring cells. EdU was added to culture medium at 5 DIV for 24hr, followed by a switch of culture medium as described in **(A)**. Scale bar: 20 μm. **(F)** i, Co-labeling of GAD-67 (green) and VGLUT1 (red) of adult spinal cord NSPC cultures at 14 DIV showed that the majority of the cells were only VGLUT1+. (ii) Immunostaining negative control in the same culture condition as (i) at 14 DIV. Blue represents Hoechst-stained nuclei. Insets show phase contrast pictures of the same field. Scale bar: 20 μm. **(G)** Scatter plot of the fluorescent intensity of each individual cell analyzed, which showed that 63.3% of the cells were VGLUT1+ at 14 DIV; while 97.4% of the VGLUT1+ cells were negative for GAD-67 (*n* = 428 cells from two coverslips were analyzed). Red and green lines denote the “cut-off” for VGLUT1 and GAD-67 immunoreactivity, respectively.

Previous studies on PC12, adult rat dorsal root ganglion neurons, and corticospinal motor neurons have reported that neurite outgrowth involves growth factor-mediated ERK1/2 activation (Hollis et al., 2009; Hashimoto and Ishima, 2011). To investigate whether the MEK/ERK1/2 pathway, as well as the PI3K/Akt and PLCγ signaling pathways played a role in neurite outgrowth, both neurite branching and the average length of the longest neurite of MAP2+ neurons were quantified in cultures treated with the corresponding inhibitors and vehicle control (Figures 5A,B). After 6 days, the culture medium containing FGF2 was supplemented instead with neurite outgrowth-inducing factors (BDNF, GDNF, and cAMP) for 7 more days (except for the NB-A/B27 control condition in which no exogenous growth factor was added) (Figure 5A). By 13 DIV, added growth factors doubled the number of neurite branches in DMSO (vehicle) control from 4.0 to 7.9 branches per cell; whereas the number of branches in NB-A/B27 control with no added growth factor remained similar to that measured at 6 DIV (4.6 vs. 4.0 branches per cell) (Figure 5C; dotted line indicates level at 6 DIV). Hence, the addition of exogenous factors (i.e., BDNF, GDNF, and cAMP) indeed promoted further neuronal differentiation of MAP2+ neurons, as indicated by the further increase in neurite outgrowth (compare Figure 2B with Figure 5B). We found that neurite branching of MAP2+ neurons was significantly suppressed when activation of ERK1/2 and PLCγ was blocked (5.2 and 6.1 branches per cell, respectively) but not when activation of Akt was blocked (7.7 branches per cell). It is interesting to note that while neurite branching was ERK1/2- and PLCγ-dependent, the inhibition of these two signaling pathways in turn resulted in an increase in neurite length of the MAP2+ neurons (from 91 μm at 6 DIV to 130.4 μm and 125.4, respectively) (Figure 5C). The ability of these MAP2+ cells to extend neurites also indicates that neurite elongation of these cells is not ERK1/2- and PLCγ-dependent. The expression of synaptophysin in MAP2+ neurons indicates that those MAP2+ neurons in the control condition began to establish synapses with neighboring cells after 2 weeks of culture and were therefore more differentiated (Figure 5D). By adding EdU to the culture medium at 5 DIV for 24 h, followed by a switch to the medium with neurite-inducing factors for 7 days (as in Figure 5A), we found that some of the MAP2+ cells with processes that came in contact with neighboring MAP2+ cells were also EdU+, once again confirming that they were newly generated neurons (Figure 5E). Co-labeling of GAD-67 and VGLUT1 revealed that 63.3% of the cells were only VGLUT1+ at 14 DIV, while 97.4% of the VGLUT1+ cells were negative for GAD-67 (*n* = 428 cells from two coverslips) (Figures 5F,G). This suggests that many of the cells in culture remained glutamatergic since 6 DIV (Figure 2E).

## Discussion

Since the role of the MEK/ERK1/2, PI3K/Akt, and PLCγ signaling pathways in adult spinal cord NSPCs has not been established, we assessed the contribution of these pathways to the proliferation and neuronal differentiation of NSPCs derived from adult rat spinal cord cultures. Proliferation was evaluated using the thymidine analogue, EdU; while neuronal differentiation was demonstrated through a time course of MAP2 staining and neurite outgrowth, as well as by the appearance of synaptophysin in the neurites. First, our data demonstrate that both the MEK/ERK1/2 and PI3K/Akt pathways are essential for proliferation of adult spinal cord NSPCs. Second, we show that the activation of ERK1/2 is necessary for directing adult spinal cord NSPCs toward a neuronal fate. Finally, our data indicate that neurite branching is dependent on the activation of the MEK/ERK1/2 and PLCγ pathways.

Currently there are only few publications that describe the stem cell niche in the adult spinal cord (Sabourin et al., 2009; Hugnot and Franzen, 2011). Among those studies, cells that express the immature neuronal marker, DCX were detected throughout the ependymal region in the adult spinal cord, where occasional proliferating nestin+ cells were also found (Hamilton et al., 2009; Sabourin et al., 2009; Hugnot and Franzen, 2011). In addition, proliferating NG2+ cells found throughout the parenchyma in the adult spinal cord have been reported to coexpress DCX (Shechter et al., 2010), even though conventionally NG2 is a marker for oligodendrocyte progenitor cells. Together with *in vivo* studies which describes coexpression of NG2 and DCX in the adult neocortex (Tamura et al., 2007) and in the adult piriform cortex (Guo et al., 2010), these findings suggest that at least a subpopulation of NG2 cells can be less committed to the oligodendrocyte lineage than previously thought and has the potential to give rise to neurons.

Nestin, a NSPC marker (Lendahl et al., 1990; Johansson et al., 1999; Namiki and Tator, 1999; Fu et al., 2005), was used in this study to characterize the expansion of adult spinal cord NSPCs in culture medium containing FGF2. We showed that by 4 DIV 50% of the cells were nestin+ and 30% were MAP2+. Based on the experiments done at early time points (at 0–3 DIV), the remaining population of the cells are most likely NG2, DCX, and/or SSEA-1 cells. The presence of cells expressing various progenitor markers (nestin, NG2, and SSEA-1) indicate the presence of different uncommitted progenitor cells with a range of potentials, and hence, the term “neural stem/progenitor cells (NSPCs).” Moreover, we can infer from our data that by 14 DIV essentially all of the NSPCs in our study had undergone neuronal differentiation as suggested by the expression of the mature neuronal marker, MAP2, as well as synaptophysin and VGLUT1.

With the use of proliferative markers, studies in blastocyst-derived embryonic stem cells (ESCs) (Li et al., 2007) and in primary cultures of embyronic (E12.5-E13.5) cortical progenitor cells (Barnabe-Heider and Miller, 2003) indicate that the activation of Akt, but not ERK1/2, is required for proliferation. In contrast, the activation of either Akt or ERK1/2 has been shown to promote proliferation in adult hippocampal (Peltier et al., 2007; Ma et al., 2009) and in adult subventricular zone (Torroglosa et al., 2007; Lao et al., 2013) neural stem cell cultures. Similarly, in adult spinal cord NSPC cultures, we have shown that the inhibition of either the PI3K/Akt pathway or the MEK/ERK1/2 pathway, but not the PLCγ pathway, markedly reduced cell division (proliferation). Past studies using different cell lines and primary neurons have suggested that cell survival involves the activation of these two pathways (Xia et al., 1995; Kennedy et al., 1997; Xue et al., 2000; Yamaguchi and Wang, 2001). Nonetheless, we have shown that the inhibition of either the PI3K/Akt pathway or the MEK/ERK1/2 pathway did not induce additional cell death in the adult spinal cord cultures when compared to the vehicle control. Hence, the reduction in cell proliferation when each of these two pathways was inhibited was not due to apoptosis. In other words, both the PI3K/Akt and MEK/ERK1/2 pathways are crucial for inducing cell proliferation of adult spinal cord NSPCs.

In the adult brain, the hippocampal subgranular zone (SGZ) and the subventricular zone (SVZ) contain at least two populations of stem/progenitor cells that are nestin+ (Doetsch et al., 1997; Fukuda et al., 2003). Type 1 and B cells (in the SGZ and SVZ, respectively), which are also GFAP+ (glial fibrillary acidic protein); and type 2 and C cells (in the SGZ and SVZ, respectively) which are GFAP- and occasionally DCX+. Type 1 and B cells, respectively, give rise to Type 2 and C cells (Suh et al., 2009). The proliferation of both of these cell types involves Akt and ERK1/2 pathways (Lao et al., 2013). However, in the adult spinal cord, the populations of stem/progenitor cells are much less well-characterized; they appear to be located at the dorsal area of the ependymal zone in which proliferating cells, nestin+ cells, GFAP+ cells, and BLBP+ (brain-lipid-binding protein) cells have been detected (Shechter et al., 2007; Meletis et al., 2008; Hamilton et al., 2009; Sabourin et al., 2009). In this area there are also DCX+ cells (Shechter et al., 2007). Additional characterization is required to determine the number and type of progenitor populations in the adult spinal cord, as well as how these populations interact. Our cultures were enriched for NSPCs, but we cannot rule out the presence of other adult spinal cord cells particularly at early DIV. However, we found that by 14 DIV all the cells were MAP2+, indicating that all of the adult spinal cord NSPCs present in the culture underwent neuronal differentiation.

Collectively, these findings suggest that while the PI3K/Akt pathway may be distinctly pivotal for proliferation of ESCs and embryonic cortical progenitor cells, proliferation of adult neural stem and progenitor cells involves both the PI3K/Akt and MEK/ERK1/2 pathways in a non-overlapping manner (Table 1). The downstream targets of these two pathways regulating proliferation in adult spinal cord NSPCs remain to be determined. However, recent studies of proliferation of adult neural stem cells from other regions have identified additional pathways as well as crucial downstream targets of the PI3K/Akt and MEK/ERK1/2 pathways, including the Wnt signaling pathway (Gage, 2010), the interleukin/Janus kinase/signal transducers and activators of transcription pathway (Gomez-Nicola et al., 2011), mammalian target of rapamycin (Paliouras et al., 2012), and cyclin-dependent kinase inhibitor (Marques-Torrejon et al., 2013).

**Table 1:** **Contribution of ERK1/2, Akt, PLCγ to proliferation and neuronal differentiation of stem/progenitor cells**.

**Stem or progenitor cell origin**	**Proliferation**			**Neuronal differentiation**			**Publications**
	**MEK/ERK1/2**	**PI3K/Akt**	**PLCγ**	**MEK/ERK1/2**	**PI3K/Akt**	**PLCγ**	
Embryonic stem cells (human)	No	Yes	n/d	n/d	n/d	n/d	Li et al., 2007
Embryonic stem cells (mouse)	n/d	n/d	n/d	Yes	n/d	n/d	Li et al., 2006
E12.5 – E13.5 cortical progenitors (mouse)	No	Yes	n/d	Yes	n/d	n/d	Barnabe-Heider and Miller, 2003
Adult hippocampal neural progenitor cells (rat)	No	Yes	n/d	n/d	Inhibitory	n/d	Peltier et al., 2007
Adult hippocampal neural progenitor cells (rat)	Yes	n/d	n/d	Inhibitory	n/d	Yes	Ma et al., 2009
Adult SVZ neurospheres (mouse)	n/d	Yes	n/d	n/d	n/d	n/d	Torroglosa et al., 2007
Adult SVZ neurospheres (mouse)	Yes	Yes	n/d	n/d	n/d	n/d	Lao et al., 2013
Adult spinal cord progenitor cells (rat)	Yes	Yes	No	Yes	No	No	Present study

No: activation of the pathway is not involved.

Yes: activation of the pathway is involved.

Inhibitory: activation of the pathway is inhibitory.

n/d: not determined in the study.

With respect to neural lineage differentiation, we have shown in cultures of adult spinal cord NSPCs activation of the MEK/ERK1/2 (but not PI3K/Akt or PLCγ) pathway is required for neuronal differentiation. On the other hand, it has been reported that, in adult hippocampal neural stem cell cultures, neuronal differentiation is blocked by ERK1/2 activation and is induced through activation of PLCγ (Ma et al., 2009). Our findings provide evidence that, unlike in the adult hippocampal neural stem cells, but similar to mouse embryonic stem cells (Li et al., 2006), ERK1/2 signaling promotes neuronal differentiation in adult spinal cord NSPC cultures (Table 1). The inhibition of ERK1/2 activation likely prevents the existing/remaining adult spinal cord NSPCs from exiting cell cycle and from proceeding to differentiate into MAP2+ neurons. Although the activation of the MEK/ERK1/2 pathway has been implicated in promoting neuronal survival (Xue et al., 2000), as mentioned earlier the inhibition of the MEK/ERK1/2 pathway did not result in an increase of cell death. This implies that the decrease in the number of MAP2+ neurons we observed after ERK1/2 inhibition was not due to an increase in neuronal death but rather a suppression of neuronal differentiation. We have also shown in our NSPC cultures that the inhibition of ERK1/2 and Akt activation significantly, and to the same extent, suppressed active proliferation, yet only the inhibition of ERK1/2 activation suppressed neuronal differentiation (Table 1; Figures 3A,B). Therefore, it is unlikely that the suppression in neuronal differentiation as a result of the ERK1/2 pathway inhibition was simply due to a decrease in proliferation.

In addition to studying the role of the signaling pathways in neuronal differentiation, we have also investigated their role in neurite outgrowth in the newly generated MAP2+ cells. Our findings indicate that both the MEK/ERK1/2 and PLCγ pathways act to modulate cytoskeletal dynamics of the MAP2+ cells, as both MEK and PLCγ inhibitors, respectively, reduced neurite branching of these cells. Reminiscent of previous neurite studies (Mattson et al., 1988; Costantini and Isacson, 2000), the length of the longest neurite increases in conditions where neurite branching is compromised. These findings suggest that the MEK/ERK1/2 and PLCγ pathways may have a specific effect on neurite complexity rather than neurite elongation. During neuronal development, part of neurite outgrowth includes neurite elongation. Elongation is dependent on the dynamics of cytoskeleton, which consists of microfilaments, intermediate filaments, and microtubules (MTs). As it turns out, several microtubule-binding proteins, including MAP2, can affect the polymerization and stability of MTs (Sanchez et al., 2000). Based on previous studies a model for neurite elongation and branching has been already proposed (Hely et al., 2001; Kiddie et al., 2005). When it is phosphorylated, MAP2 has low affinity for MTs. This decrease in MAP2 association with MTs in turn decreases MT stability which results in a decrease in neurite elongation while facilitates neurite branching (Hely et al., 2001; Kiddie et al., 2005). On the other hand, when MAP2 is de-phosphorylated, this favors the binding of MAP2 to MTs, which leads to MTs bundling and polymerization and therefore promotes neurite elongation while decreases neurite branching (Hely et al., 2001; Kiddie et al., 2005). Our results, therefore, can be explained as follows. As MAP2 is a known substrate for many protein kinases, including ERKs and protein kinase C (which is activated by PLCγ), it is likely that the inhibition of ERK1/2 and PLCγ activation in fact promoted MAP2 dephosphorylation and hence its binding to MTs, resulting in an increase in neurite length (elongation) and a decrease in neurite branching.

By comparing the findings in our study with those in others, there is a recurring theme that the effect of signaling pathway activation is cellular context-dependent (Schlessinger, 2000). Along these lines, it is worth mentioning that while it has been suggested that the PI3K/Akt pathway can antagonize the MEK/ERK1/2 pathway (Mason, 2007), and vice versa (in NIH3T3 cells) (Hayashi et al., 2008), we do not have any evidence that supports such crosstalk between this two pathways in our enriched adult spinal cord NSPC culture. For instance, from our Western blot results, we did not detect the potentiation of Akt phosphorylation (vs. DMSO control) in response to ERK1/2 inactivation, or vice versa. Collectively, these data indicate that even well-established pathways warrant further investigation so as to understand the signaling mechanism that is unique to the cell type of interest.

As mentioned in the introduction, adult spinal cord NSPCs are stimulated to proliferate in response to injury (Johansson et al., 1999; Horner et al., 2000; Yamamoto et al., 2001; Danilov et al., 2006). This surge of cell proliferation, however, produces mostly reactive astrocytes (Johansson et al., 1999; Shihabuddin et al., 2000; Horky et al., 2006; Yang et al., 2006). There is also supporting evidence that there is an upregulation of FGF2 expression by the reactive astrocytes after spinal cord or spinal nerve injuries (Madiai et al., 2003; Qi et al., 2003), despite the lack of neuronal regeneration. Intriguingly, Miura et al. have reported partial functional recovery in adult rats infected with a constitutively active form of MEK1 (activator of ERK) following spinal cord injury (Miura et al., 2000). While they attributed the functional recovery to axonal regeneration, neurogenesis of the endogenous spinal cord NSPCs was not examined. Based on our study, it is likely that the constitutively active MEK/ERK signaling pathway may have also promoted neuronal differentiation of those endogenous NSPCs, resulting in the replenishment of lost neurons following injury to the spinal cord. As we gain more knowledge regarding the regulation of NSPCs in the adult spinal cord, more research to better understand such a complex system is necessary to facilitate future attempts to manipulate these NSPCs in pathological conditions. To conclude, we found in our study that the activation of the MEK/ERK1/2 pathway and the PI3K/Akt pathway (but not the PLCγ pathway) are essential for proliferation of adult rat spinal cord NSPCs; and that the activation of the MEK/ERK1/2 pathway (but not the PI3K/Akt or the PLCγ pathway) is required for neuronal differentiation of the adult spinal cord NSPCs. Together with other studies, our findings reinforce the idea that the contribution of signaling pathway activation in proliferation and neuronal differentiation is unique in different cell types and is dependent on the source of NSPCs.

## Materials and methods

### Primary cultures

All animals were used in accordance with guidelines approved by the New York University Langone Medical Center Institutional Animal Care and Use Committee. Adult male sprague-Dawley rats weighing 260–390 g were used. Animals were anesthetized and perfused with artificial cerebrospinal fluid (CSF) as described previously (Montoya et al., 2009). After perfusion and decapitation of the animal, a 20 gauge needle and syringe filled with 10 mL of cold CSF was inserted into the sacral canal to extrude the spinal cord. Microdissection of the spinal cord into approximately 1 mm^3^ pieces was carried out in cold hibernate A (HA) (Brain Bits, Springfield, IL) supplemented with 2% B27 [self-prepared; (Brewer et al., 1993)], GlutaMAX (0.5 mM), penicillin (100 U/mL) and streptomycin (100 μ g/mL) (all purchased from GIBCO, Carlsbad, CA). The dissected spinal cord tissue was digested in papain (36 U/mL, Worthington, Lakewood, NJ) and DNase I (0.02% w/v, Worthington) in a 15-mL conical tube for 5 min at room temperature (RT), then at 30°C for 25 min. The digested tissue was kept on ice and allowed to settle by gravity (~1 min). Supernatant was discarded and the pellet was resuspended with 2 mL of HA containing 0.02% DNase I. Using three fire-polished glass pipettes of decreasing diameter, the cell suspension was triturated with each no more than ten times. Before switching to another pipette, the suspension was allowed to settle (as above), and the supernatant was kept in another conical tube, while 2 mL of fresh HA (containing 0.02% DNase I) was added to the suspension. At the end of the trituration procedure when all three glass pipettes were used, a cell suspension with a total volume of 6 mL was layered on top of 5 mL of 6% OptiPrep (Accurate Chemical and Scientific Corporation, Westbury, NY) gradient in a 50-mL conical tube. The gradient was centrifuged at 822 × g for 13 min at 4°C. After centrifugation, the white myelin layer and the top half of the supernatant were discarded. After gentle mixing, the suspension was brought up to 20 mL with HA and was allowed to slowly pass through a 70 μm nylon mesh. The cell suspension was spun one last time at 480 × g for 5 min. The isolation protocol yielded ~1% of the total cell number (~110,000 cells isolated) in the spinal cord, which was calculated based on the DNA content (PureLink Genomic DNA kits, K1820–01, Invitrogen, Carlsbad, CA) of the final pellet and previous studies of amount of DNA per cell (Santen and Agranoff, 1963) and total cell number in the adult rat spinal cord (Bjugn, 1993). The final pellet was resuspended with 400 μ L of NB-A/B27 [Neurobasal A (GIBCO) supplemented with 2% B27, GlutaMAX (0.5 mM), penicillin (100 U/mL) and streptomycin (100 μ g/mL)]. Ten microliter of cell suspension (~3000 cells) was plated on poly-D-lysine-coated glass coverslips (50 μ g/mL, P6407, Sigma-Aldrich, St. Louis, MO) in 4-well plates. After 1 h in 5% CO_2_, 37°C, 500 μ L of pre-warmed NB-A/B27 containing 20 ng/mL of FGF2 (13256-029, Invitrogen) was added to each well. Adult spinal cord cultures were treated with 10 μ M U0126 (Cat#662005), 10 μ M LY294002 (Cat#440202), 2.5 μ M U73122 (Cat#662035; all from Calbiochem, San Diego, CA), or vehicle control DMSO (dimethyl sulfoxide, D2650, Sigma-Aldrich). For longer culture period, NB-A/B27 from 6 to 13 days *in vitro* were supplemented with recombinant human brain-derived neurotrophic factor (BNDF, 1 ng/mL, 10909010, Invitrogen), recombinant human glial-derived neurotrophic factor (GDNF, 0.1 ng/mL, 10907012, Invitrogen), and membrane permeable cAMP analog [8-(4-chlorophylthio)adenosine 3',5'-cyclic monophosphate sodium salt, 125 μ M, C3912, Sigma-Aldrich]. Culture medium was changed every other day in all experiments.

In Western blots, the PLC inhibitor U73122 inhibited PLCγ 1 activation at 10 μ M in our cultures. Due to its cytotoxic effect in longer exposure (6 DIV), the concentration of U73122 was eventually lowered to 2.5 μ M. At 2.5 μ M, U73122 still significantly and specifically inhibited PLCγ 1 activation while it did not induce a significant increase in cell death as compared to the vehicle control (see results). This U73122 concentration is similar to its reported IC_50_ value of 1–2.1 μ M (product information, Cat#662035 Calbiochem). The MEK inhibitor U0126 was tested at various concentrations in our cultures, resulting in a decrease in the number EdU+, MAP2+, and EdU+/MAP2+ cells as the concentrations of U0126 was increased (not shown). At the highest dosage we tested, U0126 (10 μ M) did not cause an increase in cell death when compared to vehicle control and its action was specific (see Western blot results), hence this concentration was used for subsequent experiments.

### Immunocytochemistry

**Figure 6:**
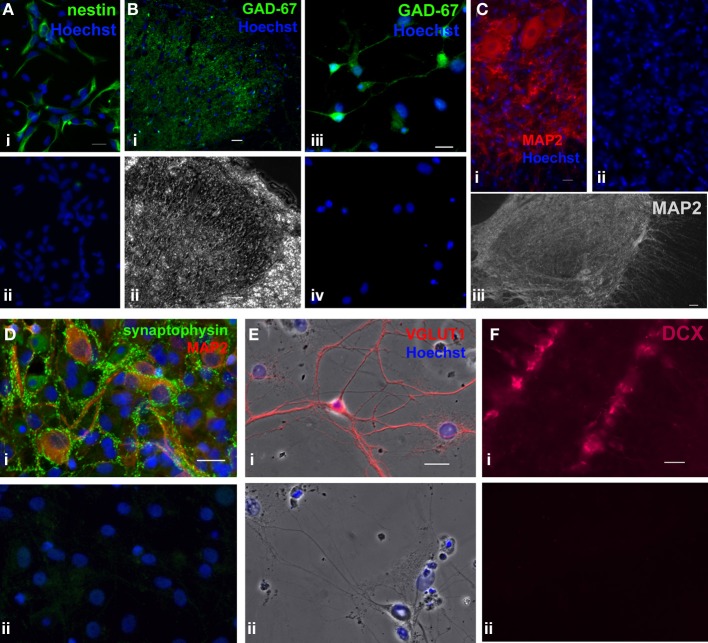
**Immunostaining controls of primary antibodies used in the study.** As positive controls, primary antibodies used in the study were tested in different cell types or tissues where they have been detected in previous studies. **(A)**, Immunostaining of mouse monoclonal nestin (green) in hippocampal progenitor cell culture from embryonic day **(E)** 18 rat embryos (1 DIV), where the majority of the cells were nestin+ (i) [previous reported in (Mistry et al., 2002)]. (ii) Negative control where the primary antibody was omitted. Scale bar: 20 μm. **(B)** i,ii, Immunostaining of mouse monoclonal GAD-67 (green) in an adult rat spinal cord section. (i) Fluorescent image showing that GAD-67 immunoreactivity was localized in lamina II of the dorsal horn, which matches the typical staining pattern of GAD-67 in the spinal cord (Edgerton et al., 2001). (ii) Phase contrast image of the same dorsal horn field. Scale bar: 20 μm. **(B)** iii,iv, Immunostaining of rat postnatal day 7 hippocampal culture (16 DIV) using mouse monoclonal GAD-67 antibody. (iii) GAD-67 staining (green). (iv) Negative control in which the primary antibody was omitted. Scale bar: 20 μm. **(C)**, Immunostaining of mouse monoclonal MAP2 in adult rat spinal cord sections. (i) MAP2 staining (red) in ventral horn neurons. Scale bar: 20 μm. (ii) Negative control where the primary antibody omitted. (iii) Composite of an adult spinal cord section taken at a lower magnification. Prominent MAP2 staining (white) was observed only in the gray matter [similar to a previous study (Suzuki-Yamamoto et al., 2000)]. Scale bar: 50 μm. **(D)**, Co-labeling of rabbit polyclonal synaptophysin 1 (green) and mouse monoclonal MAP2 (red) in rat postnatal day 7 hippocampal culture (37 DIV). (i) MAP2+ neurons formed synapses (expressed synaptophysin) with neighboring neurons. (ii) Negative control where the primary antibody was omitted. Whole molecule rabbit and mouse IgG at concentrations identical to those of the two primary antibodies were used to show specificity of the primary antibodies. Blue represents Hoechst-stained nuclei. Scale bar: 20 μm. **(E)**, Immunostaining of rabbit polyclonal VGLUT1 (red) in rat E18 hippocampal culture (22 DIV). (i) Negative control where the primary antibody was omitted. (ii) Only those cells with neuronal morphology were immunoreactive for VGLUT1. **(F)** i, Immunostaining of goat polyclonal DCX (magenta) in an adult mouse brain section. DCX+ immature neurons were detected in the dentate gyrus of the hippocampus. (ii) Negative control with the primary antibody omitted. Blue represents Hoechst-stained nuclei. Scale bar: 20 μm.

Adult rat spinal cord cells were fixed in 4% paraformaldehyde (15710, Electron Microscopy Sciences, Hatfield, PA) for 15 min and permeabilized in 0.3% Triton X-100 (X100RS, Sigma-Aldrich) [or 0.1% Tween 20 (P5927, Sigma-Aldrich) for mouse anti-stage-specific embryonic antigen 1 (SSEA-1)] for 10 min at RT. To label proliferating cells, 5-ethynyl-2'-deoxyuridine (EdU) (1.5–3 μM, from the Click-iT EdU Imaging Kit, C10337, Invitrogen), a modified thymidine analogue, was applied to the culture (for 24 h, unless otherwise stated) before fixation. To detect EdU-labeled cells, a click-iT reaction between the Alexa Fluor 488 azide and the modified EdU alkyne was carried out according to the instructions of the company. This reaction was carried out prior to immunostaining procedures with various primary antibodies. Mouse anti-nestin (~1 μg/mL, Rat-401-concentrate, Developmental Studies Hybridoma Bank, Iowa City, IA; 2.5 μ g/mL, MAB353, Millipore) and mouse anti-SSEA-1 (~1 μ g/mL, MC-480, Developmental Studies Hybridoma Bank) were used as markers for undifferentiated adult neural stem/progenitor cells. Rabbit anti-NG2 chondroitin sulfate proteoglycan (0.67 μ g/mL, AB5320, Millipore) to label neural progenitor cells. Primary antibodies used to label neurons were mouse anti-microtubule associated protein-2a and -2b (0.4–0.67 μ g/mL, MAP2, clone AP20, MAB3418, Millipore, Temecular, CA), goat anti-doublecortin (0.8 μ g/mL, DCX, sc-8066, Santa Cruz Biotechnology, Santa Cruz, CA), mouse anti-glutamic acid decarboxylase (0.67 μ g/mL, GAD-67, MAB5406, Millipore), and rabbit anti-vesicular glutamate transporter 1 (1:2000 polyclonal antiserum, VGLUT1, 135302, Synaptic Systems, Germany). Rabbit anti-synaptophysin 1 (1:150 polyclonal antiserum, 101002, Synaptic Sytems) was used to identify synapse formation. Ten percent normal goat serum (G9023, Sigma-Aldrich) was used to block non-specific binding. Cultures were incubated overnight at 4°C with primary antibodies diluted in 2% goat (or, for DCX, donkey) serum in PBS. Species-appropriate IgG, IgG1, and IgG2a conjugated with Alexa Fluor 488, 546, or 555 (1:800–1:2000, Molecular Probes, Eugene, OR) were used as secondary antibodies. After discarding the buffer containing the primary antibodies and extensive rinsing (at least 5 rinses, 5 min each), the secondary antibodies were added and incubated for 1 h at RT. Cells were counterstained with Hoechst 33342 for 15 min (6 μ g/mL). Coverslips were mounted with Aqua-Poly/Mount (Polysciences, Warrington, PA). Negative controls were prepared by omitting the primary antibodies. Rabbit and mouse whole molecule IgG (Jackson Immuno Research, West Grove, PA) at comparable concentrations to primary antibodies used for immunostaining were also used as alternative negative controls. Appropriate cell types or tissues were used as immunostaining controls to test the specificity of the primary antibodies used for immunocytochemistry in this study (Figure 6).

For TUNEL labeling, adult spinal cord cells were cultured for 4 days in FGF2-containing NB-A/B27, treated with each of the inhibitors (or DMSO), and fixed as described above. To prevent apoptosis, 20 μ M Z-VAD-FMK (Cat#219007, EMD Biosciences, San Diego, CA), a general caspase inhibitor, was used. Click-iT TUNEL Alexa Fluor 488 Imaging Assay (C10245, Invitrogen) was used to detect apoptotic cells. The TUNEL reaction, based on a copper catalyzed reaction between the Alexa Fluor azide and a modified dUTP alkyne, was carried out according to the company manual. DNase I was provided in the company kit as a positive control of the TUNEL assay.

### Immunoblotting and immunoprecipitation

Adult spinal cord cells were cultured with FGF2 for 6 days in 35 mm Petri dishes. Cells were rinsed with NB-A/B27 four times, once every hour for 4 h to remove traces of FGF2, followed by a 1 h-pre-treatment with one of the following inhibitors, 10 μ M U0126, 10 μ M LY294002, 2.5 μ M U73122, or DMSO. Cells were then exposed to FGF2 (20 ng/mL) for 30 min after which the medium was discarded and the cells were rinsed once with ice-cold PBS. The following steps were carried out on ice or at 4°C: cells were lysed directly in Petri dishes by adding 50μl of lysis buffer: RIPA (20188, Millipore) supplemented with 0.1% (v/v) SDS and 1X phosphatase inhibitor cocktail (78420, Thermo Scientific, Rockford, IL). Solubilized cell lysate was collected to one side of the dish by scraping. Lysate was then transferred to Eppendorf tubes and rocked for 5 min. Supernatant from each sample was collected after a 13-min spin at 13k rpm. Total protein concentrations were determined using the Lowry assay. Various volumes of samples containing 50 μ g of total protein were lyophilized and resuspended with 20 μ L of sample buffer. After boiling the samples for 5 min, proteins were separated by SDS-PAGE and transferred to PVDF membranes (Amersham, UK) for 3 h (100 V). Membranes were blocked in 5% BSA in Tris-buffered saline, 0.1% Tween 20 (TBST, all from Sigma-Aldrich) for 1 h at RT, and then they were incubated overnight at 4°C with primary antibodies diluted in 4% BSA in TBST. Membranes were first probed with antibodies against phospho-proteins. Then the membranes were stripped (Restore Plus, 46430, Thermo Scientific) and reprobed with antibodies against total proteins. Primary antibodies used were mouse anti-phospho-p44/42 MAPK (ERK1/2) (1:2000, Thr202/Tyr204, 9106, Cell Signaling Technology, Danvers, MA), rabbit anti-phospho-Akt (1:1000, Ser-473, 9271, Cell Signaling), rabbit anti-phospho-PLCγ 1 (1:700, Thy-783, 2821, Cell Signaling Technology), rabbit anti-p44/42 MAPK (1:1000, 9102, Cell Signaling Technology), mouse anti-Akt (1:1000, 610860, BD Biosciences), and mouse anti-PLCγ 1 (1:300, sc-7290, Santa Cruz Biotechnology). Membranes were incubated with HRP-conjugated goat anti-rabbit (1:5000, sc-2004, Santa Cruz Biotechnology) or anti-mouse (1:5000, sc-2005, Santa Cruz Biotechnology) secondary antibodies diluted in 2.5% BSA in TBST. Membranes were developed with SuperSignal chemiluminescent substrates (West Pico, 34077, West Dura, 37071, Thermo Scientific). Blots were quantified using the gel analyzer function in the ImageJ software.

For immunoprecipitation, anti-PLCγ-coated Dynabeads (Dynabeads Protein G, 100.03D, Invitrogen) was incubated with solubilized cell lysate containing 40 μ g of total protein overnight at 4°C. The lysate-antibody-Dynabeads mixture was washed three times with cold PBS and then resuspended with 20 μ L of sample buffer for subsequent Western blot analysis as described previously. Monoclonal phosphotyrosine antibody (1:1500 pTyr, PY99, sc-7020) was purchased from Santa Cruz Biotechnology.

### Image acquisition and analysis

Fluorescent images were captured using Carl Zeiss Axiovert 200 M. For each experiment, ten (using a 40× objective) to twenty (using a 20× or 40× objective) random fields of each of the coverslips in each treatment were acquired. For total cell numbers, Hoechst-stained nuclei were counted using a 10× objective over the entire coverslip. The exposure times of the fluorescent channels (DAPI, GFP, and Rhodamine) was determined using negative controls where either the primary antibody was omitted or the whole molecule IgG of matching species was added in place of the primary antibody. The fluorescent intensities of individual cells were analyzed using the AxioVision software. Cells were scored as immune-positive when their intensities were at least three standard deviations above the average values of their corresponding negative controls. Alternatively, the background fluorescence from each negative control was subtracted from the cells and fluorescence above the background was considered as positive based on the RGB histogram generated by AxioVision. All results are represented as mean + SEM. Statistical significance was determined by unpaired *t*-test when comparing two groups. When comparing three or more groups One-Way ANOVA was used. Bonferroni's multiple comparison post-test was used when comparing three to five groups and Dunnett's for five or more groups. ^*^P < 0.05, ^**^P < 0.01, ^§^*P* < 0.005, ^†^*P* < 0.001, ^#^*P* ≤ 0.0001.

### Conflict of interest statement

The authors declare that the research was conducted in the absence of any commercial or financial relationships that could be construed as a potential conflict of interest.
